# 胸腔镜与开胸肺叶切除术治疗非小细胞肺癌近期疗效的系统评价

**DOI:** 10.3779/j.issn.1009-3419.2012.07.06

**Published:** 2012-07-20

**Authors:** 雪飞 张, 小男 史, 彪 韩

**Affiliations:** 1 730000 兰州，兰州大学第一临床医学院 The First Clinical Medical College of Lanzhou University, Lanzhou 730000, China; 2 730000 兰州，兰州大学第一附属医院胸外科 Department of Thoracic Surgery, the First Hospital of Lanzhou University, Lanzhou 730000, China

**Keywords:** 肺肿瘤, 胸腔镜肺叶切除术, 开胸肺叶切除术, *Meta*分析, 系统评价, Lung neoplasms, Video-assisted thoracoscopic surgery lobectomy, Thoracotomy lobectomy, *Meta* analysis, Systematic review

## Abstract

**背景与目的:**

肺癌是临床上常见的恶性肿瘤，本研究系统评价胸腔镜与开胸肺叶切除术治疗非小细胞肺癌的近期疗效。

**方法:**

通过计算机检索Cochrane Library、Embase、Pubmed、CBM、CNKI、VIP等数据库，收集有关胸腔镜与开胸肺叶切除术治疗非小细胞肺癌的随机对照研究，由两名评价者独立评价纳入研究的质量并提取资料，用RevMan 5.0软件进行*meta*分析。

**结果:**

共纳入5篇随机对照研究，共537例患者，*meta*分析结果显示：胸腔镜与开胸肺叶切除术治疗非小细胞肺癌相比，两组手术时间（SMD=0.27, 95%CI: 0.10-0.44）、胸腔引流量（SMD=-0.23, 95%CI: -0.45–-0.01）、术后住院时间（SMD=-0.25, 95%CI: -0.46–-0.05）、并发症发生率（SMD=0.51, 95%CI: 0.32-0.80）等方面的差异有统计学意义；而两组术中淋巴结清扫个数（SMD=-0.01, 95%CI: -0.22-0.19）的差异无统计学意义。

**结论:**

胸腔镜肺叶切除术与开胸肺叶切除术相比，治疗非小细胞肺癌的淋巴结清扫个数相当，但在术中出血量、手术时间、胸腔引流液量、术后住院时间等方面有差异。

## Abstract (en)

**Background and objective:**

Lung cancer is a common malignant tumor. The aim of this study is to assess the short-term effect of video-assisted thoracoscopic surgery lobectomy and thoracotomy lobectomy in the treatment of non-small cell lung cancer.

**Methods:**

Cochrane Library, Embase, Pubmed, CBM, CNKI, VIP, and so on, were searched by computer. Randomized controlled trials comparing video-assisted thoracoscopic surgery lobectomy and thoracotomy lobectomy in the treatment of non-small cell lung cancer were collected. Two reviewers independently assessed the quality of included studies and extracted data. We analyzed the data using Review Manager (Version 5.0).

**Results:**

Five randomized controlled trials totaling 537 patients were included. The results of *meta* analysis were as follows: statistically significant difference was found in operating time (SMD=0.27, 95%CI: 0.10–0.44), chest tube drainage flow (SMD=-0.23, 95%CI: -0.45–-0.01), postoperative hospital stay (SMD=-0.25, 95%CI: -0.46–-0.05), and postoperative complications (SMD=0.51, 95%CI: 0.32–0.80). No statistically significant difference was found in the harvested lymph nodes (SMD=-0.01, 95%CI: -0.22–0.19) between video-assisted thoracoscopic surgery lobectomy and thoracotomy lobectomy in the treatment of non-small cell lung cancer.

**Conclusions:**

Compared with thoracotomy lobectomy in the treatment of non-small cell lung cancer, the amount of harvested lymph nodes was almost the same, but the amount of blood loss, operating time, chest tube drainage flow, and postoperative hospital stay were different.

肺癌是临床上常见的恶性肿瘤，死亡率高^[1]^。我国肺癌的新发病例也在增加^[2]^，其中非小细胞肺癌（non-small cell lung cancer, NSCLC）约占全部肺癌的80%，是肺癌最常见的类型，传统肺癌的外科治疗方式为开胸手术（thoracotomy lobectomy, TL）。随着电视辅助胸腔镜手术（video-assisted thoracoscopic surgery, VATS）的引进，因其具有创伤小、术后疼痛轻、肺功能损伤轻、恢复快，且能用于不能耐受TL的患者而迅速得到广泛应用^[3]^，但与传统TL相比，是否具有相同的治疗效果或能改善术后生活质量或增加手术并发症没有明确的依据。因此，本文采用系统评价的方法进行分析，以期为临床实践提供参考依据。

## 1 资料与方法

### 1.1 纳入与排除标准

#### 1.1.1 研究对象

纳入标准：①原发性NSCLC患者，CT示无肺门和纵隔淋巴结转移；②之前未接受手术治疗；③种族、国籍、年龄、性别不限；④无明显手术禁忌症，肝肾功、血液学、心电图无明显异常。伴有严重内科疾患及感染者、同时患第二个恶性肿瘤者均被排除。

#### 1.1.2 研究类型

随机对照研究（randomized controlled trial, RCT），无论是否采用盲法和分配隐藏。

#### 1.1.3 干预措施

VATS（观察组）*vs* TL（对照组）。

#### 1.1.4 结局测量指标

手术时间、胸腔引流量、并发症发生率、术中出血量、术中淋巴结清扫个数、引流管放置时间、术后住院时间。

### 1.2 文献检索

计算机检索中国生物医学文献数据库（CBM，1978年-2012年3月）、中文科技期刊数据库（VIP，1989年-2012年3月）、中国期刊全文数据库（CNKI，1994年-2012年3月）、中华医学会数字化期刊（1998年-2009年）、万方数据库（1980年-2012年3月）、Cochrane library、Pubmed（1966年-2012年3月）、Embase（1974年-2012年3月及相关临床试验网站；并运用Google、Medical Martix等搜索引擎查找相关参考文献及灰色文献。主要检索词：“Video-assisted thoracoscopic surgery”、“VATS”、“thoracotomy”、“lobectomy”、“lung cancer”、“胸腔镜手术”、“开胸手术”、“肺癌”。

### 1.3 文献筛选和资料提取

两名研究人员独立地对符合纳入标准的试验进行资料提取，填写资料提取表格，并交叉核对提取的资料，如遇意见不一致双方讨论解决或由第三者判断，缺乏的资料通过与临床试验人的负责人联系予以补充。提取的信息包括：作者、出版年、样本量、研究对象、研究设计、手术时间、胸腔引流量、并发症发生率、术中出血量、术中淋巴结清扫个数、引流管放置时间、术后住院时间。

### 1.4 质量评价

纳入研究的方法学质量评价按照Cochrane系统评价手册4.2.2版中关于随机对照试验的4条质量评价标准进行：①随机方法是否正确；②随机分配隐藏方案是否正确；③是否采用盲法，对哪些人采用盲法；④有无失访或退出。依据以上评价指标，将研究质量从高到低分为A、B、C三级，其中A级为低度偏倚，B级为中度偏倚，C级为高度偏倚。

### 1.5 统计分析

采用国际Cochrane协作组提供的RevMan 5.0软件进行*meta*分析，计量资料采用标准化均数差（standard mean difference, SMD）；计数资料采用风险比（risk ratio, RR）为疗效分析统计量，各效应量均以95%可信区间（confidence interval, CI）表示，并绘制森林图。纳入研究同质性（*I^2^* < 50%, *P* > 0.1）好则采用固定效应模型分析，反之则采用随机效应模型分析。若异质性过大则采用描述性分析。必要时采用敏感性分析检验结果的稳定性。对于无法合并的指标采用描述性分析。

## 2 结果

### 2.1 文献检索结果

最初检索到文献2, 670篇，通过阅读题名和摘要排除2, 655篇非RCT临床研究和动物实验，初步纳入研究15篇，阅读全文排除不符合纳入标准的研究10篇，最终纳入RCT 5篇^[4-8]^（检索流程见图 1）。5项研究共收集病例537例，VATS组265例，TL组272例，各研究均比较了患者年龄、性别、体能状态等基线情况，结果显示试验组和对照组间基线可比性较好（*P* > 0.05）。纳入研究的基本特征见表 1。

**1:**
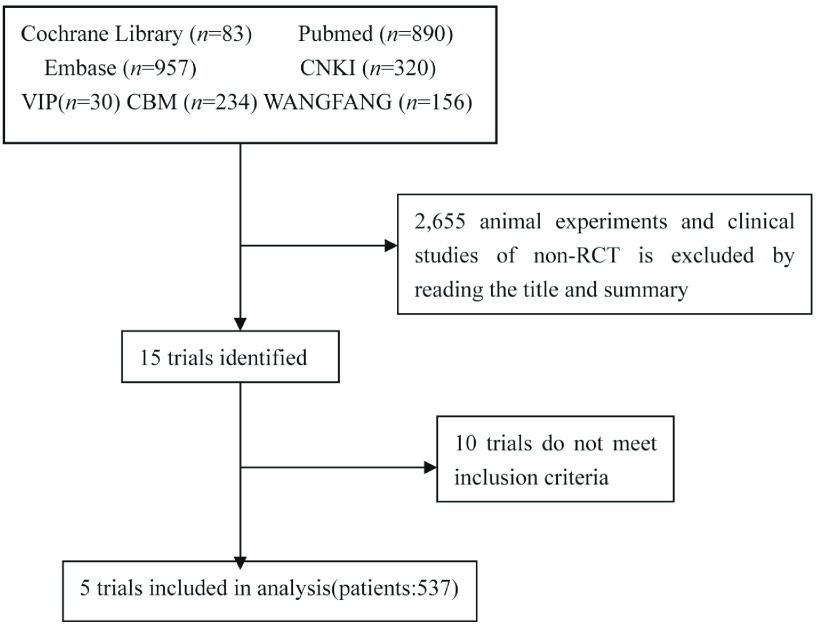
纳入研究流程图 Selection of trials

**1:** 各项纳入研究的基本特征 Assessment characteristics of included studies

Included studies	Study sites	Cases		Male/Female (*n*/*n*)		Age (year)		Follow-up time
		VATS	TL	VATS	TL	VATS	TL	
Zhang 2010^[4]^	China	133	138	66/67	66/72	56±8	56±8	> 1 year
Long 2008^[5]^	China	22	22	15/7	14/8	58.3±9	56.7±12	No-mention
Kirby 1995^[6]^	America	25	30	10/15	14/16	58±9	62±12	13 months
Craig 2001^[7]^	England	22	19	8/14	14/5	47-74	46-78	18 months
Liu 2010^[8]^	China	63	63	41/22	43/20	51-78	53-81	1 year
VATS: video-assisted thoracoscopic surgery; TL: thoracotomy lobectomy.											

### 2.2 纳入研究的方法学质量评价

文献质量评价按Cochrane系统评价手册4.2.2版中有关RCT的质量评价标准进行，纳入的5项研究^[4-8]^均提到随机分组，2项研究^[4, 5]^随机方法充分，2项研究^[5, 7]^分配隐藏充分，1项研究^[5]^报道了盲法，2项研究^[5, 6]^提及退出，评价结果见表 2。

**2:** 纳入研究的方法学质量评价 Assessment methodologic quality of included studies

Author	Sequence generation	Allocated concealment	Blinding	Follow-up/Withdraw	Quality grading
Zhang 2010^[4]^	Yes	Unclear	Unclear	Unclear	B
Long 2008^[5]^	Yes	Yes	Yes	Yes	A
Kirby 1995^[6]^	Unclear	Unclear	Unclear	Yes	B
Craig 2001^[7]^	Unclear	Yes	Unclear	Unclear	B
Liu 2010^[8]^	Unclear	Unclear	Unclear	Unclear	B

### 2.3 *meta*分析结果

#### 2.3.1 手术时间

5项研究^[4-8]^均报道了手术时间，纳入研究间无异质性（*I^2^*=41%, *P*=0.15）*meta*分析结果（图 2）显示，VATS与TL相比，治疗NSCLC在手术时间方面差异有统计学意义（SMD=0.27, 95%CI: 0.10-0.44），VATS组的手术时间长于TL组。

**2:**
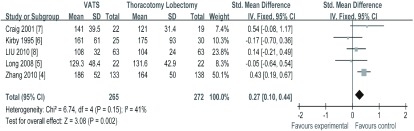
两组手术时间的比较 The comparison of the operating time

#### 2.3.2 术中出血量

3项研究^[4, 5, 8]^报道了术中出血量，由于纳入研究间存在相当大的异质性（*I^2^*=98%, *P* < 0.000, 01）不适合进行*meta*分析，故进行描述性分析。其中张轶等^[4]^的研究中VATS组术中出血量为（228±180）mL，TL组为（246±245）mL，两组差异无统计学意义。龙浩等^[5]^的研究中，VATS组术中出血量为（97.3±71.6）mL，TL组为（121.4±105.5）mL，差异无统计学意义。刘宗亮^[8]^研究报道VATS组术中出血量为（172±12）mL，TL组为（283±53）mL，两组差异有统计学意义（*P* < 0.05）。

#### 2.3.3 术中淋巴结清扫个数

3项研究^[4-6]^报道了淋巴结清扫个数，纳入研究间无异质性（*I^2^*=0, *P*=0.84）。*meta*分析结果（图 3）显示，VATS组与TL组相比，治疗NSCLC在术中淋巴结清扫个数（SMD=-0.01, 95%CI: -0.22-0.19）方面差异无统计学意义。

**3:**
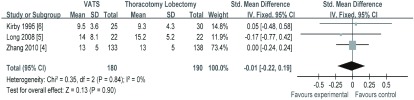
两组术中淋巴结清扫的个数 The comparison of harvested lymph nodes

#### 2.3.4 胸腔引流量

2项研究^[4, 5]^报道了胸腔引流量，纳入研究间无异质性（*I^2^*=0, *P*=0.59），*meta*分析结果（图 4）显示，VATS组与TL组相比，治疗NSCLC在术后胸腔引流量（SMD=-0.23, 95%CI: -0.45--0.01）方面差异有统计学意义，VATS组术后胸腔引流液量少于TL组。

**4:**
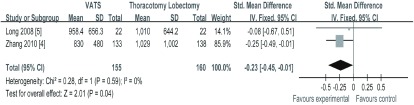
两组胸腔引流量的比较 The comparison of chest tube drainage flow

#### 2.3.5 引流管放置时间

4项研究^[4-6, 8]^报道了引流管放置时间，由于纳入研究间存在异质性（*I^2^*=95%, *P* < 0.000, 01），不适合进行*meta*分析，故进行描述性分析。其中张轶等^[4]^研究中VATS组术后引流管放置时间为（3.4±1.8）d，LT组为（3.2±2.5）d，两组差异有统计学意义（*P* < 0.05）。龙浩等^[5]^研究中VATS组术后引流管放置时间为（3.6±2.9）d，LT组为（3.0±1.5）d，两组差异无统计学意义。Kirby等^[6]^研究中VATS组术后引流管放置时间为（4.6±3.3）d，LT组为（6.5±4.8）d，两组差异无统计学意义。刘宗亮^[8]^研究报道VATS组术后引流管放置时间为（2.3±1.6）d，LT组为（4.7±1.2）d，两组间差异有统计学意义（*P* < 0.05）。

#### 2.3.6 术后住院时间

4项研究^[4, 6-8]^报道了术后住院时间，但由于刘宗亮^[8]^研究报道不充分，文献质量偏低而排除，其它3篇文献符合*meta*分析数据合并标准（*I^2^*=28%, *P*=0.25），采用固定效应模型进行分析。*meta*分析结果（图 5）显示，VATS组与TL组相比，治疗NSCLC在术后住院时间（SMD=-0.25, 95%CI: -0.46--0.05）方面差异有统计学意义，VATS组术后住院时间缩短。

**5:**
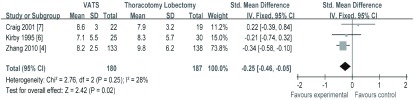
两组术后住院时间的比较 The comparison of postoperative hospital stay

#### 2.3.7 术后并发症

3篇研究^[4-6]^报道了术后并发症，纳入研究间无异质性（*I^2^*=0, *P*=0.84），*meta*分析结果（图 6）显示，VATS组与TL组相比，治疗NSCLC在术后并发症（SMD=0.51, 95%CI: 0.32-0.80）方面差异有统计学意义，VATS组手术并发症少于TL组。

**6:**
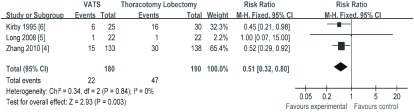
两组术后并发症的比较 The comparison of postoperative complications

#### 2.3.8 发表偏倚

对纳入文献进行漏斗图分析。漏斗图对称性较好，提示发表偏倚的可能性较小（图 7）。

**7:**
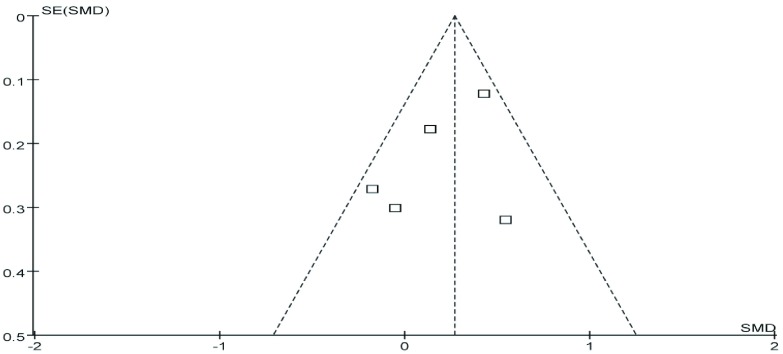
5篇文献的漏斗图分析 Funnel plot analysis of 5 literatures

## 3 讨论

手术是NSCLC的主要治疗方法，术后预后较好，主要对象为病变局限、无明显胸内器官侵犯及远道转移的Ⅰ期、Ⅱ期患者，包括部分Ⅲ期估计可以全部切除的患者。NSCLC存在微转移灶，为手术治疗成败的关键。及早发现肺癌进行手术切除和淋巴结清扫可使患者生命延长，5年生存率可达50%^[9]^。自1993年Kirby等^[10]^首次报道了应用VATS进行肺叶切除术以来，因其具有创伤小、恢复快、痛苦小及伤口美观等优点受到患者和胸外科医生的欢迎，已广泛应用于各种胸部疾病的治疗^[11]^。但与传统TL相比，胸腔镜辅助肺叶切除术的近期疗效尚不明确，本文收集了5项RCT并对两者手术时间、术中出血量、术中淋巴结清扫个数、术后胸腔引流量、引流管放置时间、术后住院时间及并发症发生率进行了*meta*分析，旨在对VATS治疗NSCLC的近期疗效做出系统评价。

本系统评价对纳入研究进行*meta*分析显示，VATS组和TL组相比进行肺叶切除术的淋巴结清扫个数无统计学差异，但手术时间延长，出血量减少，并发症发生率减低，手术时间的延长主要与当前腔镜手术器械的限制和手术者操作的熟练程度有关，随着腔镜手术的大量开展，术者熟练程度的提高，腔镜手术的速度会逐步提高。充分的淋巴结清扫是保证肺癌手术达到根治效果的关键，也是肿瘤学安全性的重要内容^[12]^，本研究显示胸腔镜手术与开胸手术清扫的淋巴结数目相当，可得到同样的根治效果。出血量的多少也影响肺癌患者的预后，出血量增多会增加输血的概率，而输血会影响患者的免疫功能，从而造成肺癌的复发转移^[13]^。根据刘宗亮^[8]^研究报道，VATS组术中出血量为（172±12）mL，LT组术中出血量为（283±53）mL，两组间差异有统计学意义（*P* < 0.05）。

*Meta*分析显示VATS组与TL组相比，具有胸腔引流液量减少、引流管放置时间缩短、术后住院时间缩短、并发症发生率降低（肺癌切除术后主要的并发症包括感染、肺不张、漏气、心律失常、肺栓塞等），同时胸腔镜手术具有切口小、肺功能损害轻、引流液量少的优点，虽目前各方面的原因导致手术时间延长，但感染等并发症仍较少。有文献^[14]^报道TL组的并发症发生率高达27.9%，远高于VATS组的17.2%，因此VATS手术能减少术后并发症的发生，同时有创伤小、恢复快的优点。Whitson等^[15]^也报道了VATS会缩短患者的住院时间，提示VATS手术的患者住院时间比TL手术短，从而大大提高了患者的生活质量。胸腔积液是细菌等感染物的良好培养基，患者胸腔引流液量的减少，减少了术后感染的发生，也有利于胸腔引流管的提前拔出，减少胸管对胸壁的刺激，也减少了医源性感染的发生。患者引流管放置时间的测定取决于引流液量的多少和医生的判断，容易受主观因素的影响，因此，胸腔引流管放置时间的判断缺乏客观性。张轶等^[4]^、龙浩等^[5]^及Craig等^[7]^检测肺癌术后机体释放的炎症介质包括C反应蛋白、IL-6、IL-8和肿瘤坏死因子（tumor necrosis factor, TNF）α等，发现术后两组患者IL-6、IL-8和IL-10的血浆浓度均有不同程度的升高，但VATS术后急性炎症反应明显轻，免疫抑制弱，减少了术后肿瘤转移、复发的机会，从而提高患者的生存率。总之，肺癌患者的预后尽管与组织类型、分化程度和病理分期等密切相关，但是手术方式也在其中发挥着一定作用。

本研究存在一定的局限性：①纳入文献只有1篇对盲法进行描述、2篇对分配隐藏进行描述，随机方法也不完全清楚，因此，存在选择性偏倚、实施偏倚、测量性偏倚的可能性；②纳入研究的病例数有限，而且部分评价指标存在异质性，造成结果的客观性受到一定影响，这有待于今后更多设计良好的多中心前瞻性随机对照研究报道，以作出更有说服力的评价；③未考虑患者种族、国籍、年龄、性别等因素对结果的影响，可能导致研究对象基线不平衡，从而可能对结果产生影响，但各研究采用了随机方法进行了分组，从而使这些因素基本均衡，排除了这些因素造成的偏倚。

综上所述，手术治疗NSCLC时VATS与TL相比，术中出血量减少、胸腔引流液量减少、引流管放置时间缩短、术后住院时间缩短、并发症发生率降低、淋巴结清扫个数相当、手术时间稍长，因此VATS治疗NSCLC的近期疗效优于TL。
